# Involvement of POMC neurons in LEAP2 regulation of food intake and body weight

**DOI:** 10.3389/fendo.2022.932761

**Published:** 2022-10-28

**Authors:** Guangpin Chu, Hualing Peng, Nana Yu, Yuejin Zhang, Xueling Lin, Yisheng Lu

**Affiliations:** ^1^ Department of Physiology, School of Basic Medicine, Tongji Medical College, Huazhong University of Science and Technology, Wuhan, China; ^2^ Institute of Brain Research, Collaborative Innovation Center for Brain Science, Huazhong University of Science and Technology, Wuhan, China; ^3^ Hubei Key Laboratory of Drug Target Research and Pharmacodynamic Evaluation, Huazhong University of Science and Technology, Wuhan, China

**Keywords:** liver-expressed antimicrobial peptide 2 (LEAP2), arcuate nucleus (ARC), food intake, energy metabolism, proopiomelanocortin (POMC)

## Abstract

Liver-expressed antimicrobial peptide 2 (LEAP2) is a newly discovered antagonist of the growth hormone secretagogue receptor (GHSR) and is considered the first endogenous peptide that can antagonize the metabolic actions of ghrelin. The effects of ghrelin administration on feeding behavior, body weight, and energy metabolism involve the activation of orexigenic neurons in the arcuate nucleus (ARC) of the hypothalamus. It is unclear, however, if LEAP2 applied directly to the ARC of the hypothalamus affects these metabolic processes. Here, we show that overexpression of LEAP2 in the ARC through adeno-associated virus (AAV) reduced food intake and body weight in wild-type (WT) mice fed chow and a high-fat diet (HFD) and improved metabolic disorders. LEAP2 overexpression in the ARC overrides both central and peripheral ghrelin action on a chow diet. Interestingly, this AAV-LEAP2 treatment increased proopiomelanocortin (POMC) expression while agouti-related peptide (AGRP)/neuropeptide Y (NPY) and GHSR levels remained unchanged in the hypothalamus. Additionally, intracerebroventricular (i.c.v.) administration of LEAP2 decreased food intake, increased POMC neuronal activity, and repeated LEAP2 administration to mice induced body weight loss. Using chemogenetic manipulations, we found that inhibition of POMC neurons abolished the anorexigenic effect of LEAP2. These results demonstrate that central delivery of LEAP2 leads to appetite-suppressing and body weight reduction, which might require activation of POMC neurons in the ARC.

## Introduction

Liver-expressed antimicrobial peptide 2 (LEAP2) is a 40-amino-acid peptide (1) identified as an endogenous antagonist (2–4) or inverse agonist (5) of the orexigenic hormone ghrelin’s receptor, growth hormone secretagogue receptor (GHSR) (6, 7). LEAP2 is mainly expressed in the liver and jejunum and secreted into the blood circulation in a metabolic status-dependent way in rodents and humans (2, 8–10). The orexigenic effects of exogenous ghrelin can be neutralized by LEAP2 (2, 5, 8, 11–15); however, whether LEAP2 could affect food intake and body weight without exogenous ghrelin is still controversial (2, 5, 8, 13, 14, 16), which might be due to differences in LEAP2 concentration and route of administration or nutritional status of the experimental animals. In addition, continuous LEAP2 treatment of calorie-restricted GHSR-knockout mice could still induce body weight loss, hypoglycemia, and body temperature reduction, implying LEAP2’s ghrelin–GHSR signaling independent effects (14).

The arcuate nucleus (ARC) is critical for energy metabolism in the hypothalamus (17–19). The anorexigenic proopiomelanocortin (POMC) neurons and orexigenic agouti-related peptide (AGRP)/neuropeptide Y (NPY) neurons are the “first-order neurons” responding to the circulating signals of hunger and satiety (17, 20, 21). Most AGRP/NPY (94%) neurons and a few POMC neurons (8%) in the ARC are GHSR positive (22, 23), and ghrelin increases food intake and adiposity by acting on GHSR-expressing AGRP/NPY neurons (24–27). LEAP2 was detected in the cerebrospinal fluid (28), and the intracerebroventricularly (i.c.v.) injected N-terminal of LEAP2 concentrated in the ARC among the nuclei of the hypothalamus (11). Although it was reported that LEAP2 inhibits AGRP/NPY neuronal activity (9), the effects after LEAP2 delivery to the ARC are still unexplored.

In the present study, we firstly investigated the effects of adeno-associated virus (AAV)-mediated overexpression of LEAP2 in the ARC of wild-type (WT) mice under metabolic conditions of chow and high-fat diet (HFD) feeding. We have also examined the effects of LEAP2 overexpression and diet on hypothalamic AGRP/NPY, POMC, and GHSR levels. We showed for the first time that administration of AAV vectors encoding LEAP2 to the ARC tempered food intake and long-term body weight gain together with elevation of hypothalamic POMC expression in mice fed chow and an obesogenic diet. Additionally, we investigated food intake, body weight, and c-Fos expression in the ARC POMC neurons after injecting the LEAP2 peptide into the third ventricle. Also, the effect of LEAP2 on POMC neuronal activity was measured by calcium imaging *in vitro*. Finally, we used the chemogenetic method to determine whether POMC neurons are required to mediate LEAP2’s effect on food intake.

## Materials and methods

### Animals

Adult male WT C57BL/6J mice (8 weeks old) were obtained from Charles River, China. POMC-Cre mice (29) were generous gifts from Prof. Guo Zhang (Huazhong University of Science and Technology, Wuhan, China). Rosa-tdTomato mice (30) were purchased from The Jackson Laboratory (007914). POMC-Cre;Rosa-tdTomato reporter mice were generated by crossing POMC-Cre with Rosa-tdTomato mice. Less than five mice were housed per cage at 22°C–23°C and 50%–55% humidity, with a 12-h light/dark cycle. Standard rodent chow (12.8% calories from fat) and high-fat (60% calories from fat) diets were purchased from Xietong Bioscience (Jiangsu, China). The Animal Care and Use Committee of Huazhong University of Science and Technology approved all experiments.

### Virus and peptide

AAV9-CMV-EGFP (AAV-GFP, 2.14 × 10^13^ vg/ml) and AAV9-CMV-LEAP2-EGFP (AAV-LEAP2, 1.86 × 10^13^ vg/ml) viruses were designed and produced by Vigene Biosciences (Jinan, China) using standard methods. rAAV-EF1α-DIO-GCaMP6m (2.64 × 10^12^ vg/ml) was constructed by BrainVTA (Wuhan, China). AAV-EF1α-DIO-hM4Di-EGFP (4.3 × 10^12^ vg/ml) was purchased from Obio Technology (Shanghai, China).

LEAP2 (38–77) (human)/LEAP2 (37–76) (mouse) was purchased from Phoenix Pharmaceuticals Inc. (075-40, Burlingame, CA, USA). The peptide was prepared at a 10-μM stock solution in water, then diluted in artificial cerebrospinal fluid (aCSF) for i.c.v. injection. Acyl-ghrelin was purchased from Taijia Biotechnology (603476, Hangzhou, China) and dissolved in 0.9% saline.

### Stereotaxic surgery

To inject AAV-GFP or AAV-LEAP2 into the ARC, WT mice were anesthetized with isoflurane (1%–3%, RWD, Shenzhen, China) and placed on a stereotaxic instrument (RWD, Shenzhen, China). The virus was bilaterally injected (300 nl per side) with a glass pipette using an automatic microinjection system (World Precision Instruments, USA) at the coordinates relative to bregma: anteroposterior, −1.7 mm; mediolateral, ± 0.23 mm; and dorsoventral, −5.8 mm, at a rate of 60 nl/min. After the injection, the glass pipette was left in place for 5–10 min and removed slowly. The skin was sutured and sterilized with iodophors. Following injections, the mice were returned to their home cages. Only body-weight–matched mice were used for virus injection and subsequent metabolic manipulations.

To deliver the LEAP2 peptide to the third ventricle through the cannula, mice were anesthetized and placed on the stereotaxic instrument. A guide cannula (RWD, Shenzhen, China) was inserted into the third ventricle at 1.7 mm posterior to bregma and 5.5 mm below the skull surface using stereotaxic coordinates. Metal bone screws and dental cement were used to secure the cannula to the skull. Animals were placed in individual cages and allowed to recover for 1 to 2 weeks prior to any further procedures.

### Food intake and body weight measurements

For AAV-injected mice, food intake was measured 3–8 weeks after the virus injection. Food pellets were weighed every day for four successive days each week, and the average food intake per day was calculated. The body weight of mice was monitored weekly.

For the i.c.v. acute administration of LEAP2 study, mice were fasted for 4 h and then received an i.c.v. injection of LEAP2 or aCSF (2 μl) at the beginning of the dark cycle (19:00). Food intake was then measured at 1, 2, 4, and 12 h after the injection. To determine the long-term effects of LEAP2 i.c.v. injection, mice received either LEAP2 (10 nM in 2 μl) or aCSF once a day for 10 days. The food intake and body weight of mice were measured daily.

### Body composition measurement

Total body fat mass and lean mass were measured by a Minispec LF50 body composition analyzer (Bruker, Germany). Epididymal white adipose tissue (eWAT), inguinal white adipose tissue (iWAT), and interscapular brown adipose tissue (iBAT) were isolated and weighed after mice were anesthetized by 1% pentobarbital sodium and sacrificed.

### Glucose tolerance test

Mice were deprived of food overnight for 16 h and received an intraperitoneal (i.p.) injection of d-glucose (2 g/kg of body weight). Blood glucose was then measured using a glucometer (OneTouch Ultra) from a tail puncture at different time points (0, 15, 30, 60, and 120 min).

### CSF preparation, brain tissue dissection, and plasma collection

CSF collection procedures were developed as previously described (31, 32). Mice were anesthetized, then mounted on a stereotaxic system (RWD, Shenzhen, China) with the head secured at a ~ 30°–45° angle facing downwards. An incision was made above the neck, and the subcutaneous muscles were separated by blunt dissection with forceps, allowing exposure of the dura mater of the cisterna magna without any bleeding. CSF was withdrawn from the cisterna magna cavity with a pulled sterilized glass capillary held by a holder. Approximately 5–10 μl of CSF was collected per mouse. CSF was kept in an Eppendorf tube on ice and spun in a cold centrifuge at 1,500 rpm for 15 min. The supernatant was stored at −80°C until use.

For the hypothalamus, tissue was dissected according to the established method (33). In brief, the hypothalamus was dissected along the anterior border of the optic chiasm, the posterior border of the mammillary body, the upper border of the anterior commissure, and the lateral border halfway from the lateral sulcus on the brain ventral side. Hypothalami were lysed with tissue lysis buffer, and protein concentrations were determined using a BCA assay kit (P0012, Beyotime, Shanghai, China).

Mice were food-restricted for 4 h before blood collection. For LEAP2 concentration analysis, mouse blood was collected from the orbital sinus into ice-cold EDTA-coated tubes, and aprotinin (final concentration 250 KIU/ml, Sigma-Aldrich, Germany) was added. Plasma was isolated by centrifugation at 3,000 rpm for 20 min at 4°C and the supernatant was transferred to new Eppendorf vials, then stored at −80°C until processing.

### LEAP2 level determination and plasma total cholesterol or triglyceride analysis

LEAP2 levels of CSF, plasma, and hypothalamic tissue samples were determined by a LEAP2 (38–77) (human)/LEAP2 (37–76) (mouse) enzyme-linked immunosorbent assay (ELISA) kit (EK-075-40, Phoenix Pharmaceuticals, Inc., Burlingame, CA, USA) following the manufacturer’s instructions. The minimum detectable concentration of LEAP2 in this assay was 0.22 ng/ml. Hypothalamic LEAP2 content was normalized to the amount of protein.

Plasma total cholesterol (TC) or triglyceride (TG) levels were measured using the corresponding assay kits (A111-1-1 for TC and A110-1-1 for TG, Jiancheng Bioengineering Institute, Nanjing, China) according to the manufacturer’s instructions.

### Hematoxylin and eosin staining

eWAT and liver tissue samples were harvested and fixed in Bouin’s solution or 4% paraformaldehyde (PFA) and embedded in paraffin. Tissues were sectioned at 5 μm thickness and stained sequentially with hematoxylin and eosin solutions. Each slide was examined, and images were collected using a brightfield microscope (Nikon Eclipse CI). The image of interest from eWAT was imported into Image-Pro Plus, and a known linear scale bar was used to set scale calibration. The adipocyte areas were depicted manually, and the size of the selected adipocyte was measured by the software. The area below 350 mm^2^ or above 15,000 mm^2^ was excluded. After the number and the size of adipocytes were determined, the frequency distribution of total adipocytes and the overall average area of the adipocytes were calculated in Microsoft Excel. These steps were repeated for each image in each mouse, and the relative frequency and adipocyte area were statistically analyzed.

### Thermography

Thermal images of mice were acquired using a calibrated infrared camera (Flir, Boston, MA). The fur covering the iBAT area was gently shaved. Freely moving mice were thermally imaged at room temperature from approximately 15:00 to 17:00. The data were analyzed and calculated with Tools+ software, and the mean values of their subcutaneous iBAT temperatures are shown.

### Chronic acyl-ghrelin administration

Mice infected with AAV-GFP or AAV-LEAP2 in the ARC were implanted with Alzet osmotic mini-pumps (model 1002, DURECT Corporation, Cupertino, CA) for 14-day continuous infusions. The mini-pumps were filled with sterilized 100 µl acyl-ghrelin or vehicle (saline), and the solution was delivered at a rate of 0.25 µl/h. For subcutaneous acyl-ghrelin treatment, pumps were implanted subcutaneously in the interscapular region to deliver continuous acyl-ghrelin infusion to achieve a cumulative daily dose of 4 mg/kg of body weight, as previously described (34). For intracerebroventricular acyl-ghrelin treatment, a cannula (0008851, Alzet brain infusion kit 3) was implanted into the lateral ventricle (anteroposterior, −0.6 mm; mediolateral, −1.3 mm; and dorsoventral, −2.3 mm), and a pump was implanted subcutaneously connected to the cannula *via* a 2-cm-long catheter. Acyl-ghrelin concentration in the pumps for intracerebroventricular infusion was 3 µg/day, as previously described (35). Food intake and body weight were measured daily for 13 days following pump implantation.

### RNA isolation and quantitative reverse transcription PCR

Tissues from the hypothalamus and iBAT were isolated on ice and stored at −80°C. Total RNA was extracted with RNAiso Plus (Code No. 9108, TaKaRa). RNA quantity and quality were determined with NanoDrop2000C spectrophotometry (Thermo Scientific). A total of 1 μg of RNA was reverse-transcribed into cDNA using the HiScript II Q RT SuperMix for quantitative reverse transcription PCR (qRT-PCR) (+ gDNA wiper) (R223-01, Vazyme, Nanjing, China). qRT-PCR was conducted on Bio-Rad CFX384 Real-Time System with ChamQ SYBR qRT-PCR Master Mix (Q311-02, Vazyme, Nanjing, China) according to the manufacturer’s recommendations. The abundance of different transcripts was assessed in triplicates. The primers used were as follows: *Leap2*, forward: 5′-CAGCTAAAACTCTTTGCAGTGC-3′, reverse: 5′-TCTCCGGGATCTCTTTGCTGA-3′; *Ucp1*, forward: 5′-AGGCTTCCAGTACCATTAGGT-3′, reverse: 5′-CTGAGTGAGGCAAAGCTGATTT-3′; *Agrp*, forward: 5′-CGGCCACGAACCTCTGTAG-3′, reverse: 5′-CTCATCCCCTGCCTTTGC-3′; *Npy*, forward: 5′-CTACTCCGCTCTGCGACACT-3′, reverse: 5′-AGTGTCTCAGGGCTGGATCTC-3′; *Pomc*, forward: 5′-GAGGCCACTGAACATCTTTGTC-3′, reverse: 5′-GCAGAGGCAAACAAGATTGG-3′; *Ghsr*, forward: 5′- TGGAGATCGCGCAGATCAG-3′, reverse: 5′- CCGGGAACTCTCATCCTTCAG-3′; *Gapdh*, forward: 5′-TGTGTCCGTCGTGGATCTGA-3′, reverse: 5′-TTGCTGTTGAAGTCGCAGGAG-3′. The final relative expression levels were computed using the 2^−ΔΔCt^ method. Data were normalized and reported as fold changes compared with the control group.

### Western blot

The hypothalamus tissue samples were isolated and homogenized in ice-cold RIPA lysis buffer (P0013B, Beyotime, Shanghai, China) supplemented with proteinase inhibitors, and then subjected to centrifugation at 12,000 rpm for 15 min at 4°C to collect the supernatant. A BCA protein assay kit (P0012, Beyotime, Shanghai, China) was used to measure protein concentration. SDS-PAGE separated proteins were transferred onto PVDF membranes (Merck Millipore). The membranes were then blocked with tris-buffered saline (TBS) containing 0.1% Tween-20 and 5% nonfat powdered milk for 1 h at room temperature, followed by overnight incubation at 4°C with primary antibodies for AGRP (1:500, ab113481, Abcam), POMC (1:2,000, 66358-1-Ig, Proteintech), GHSR (1:500, ab85104, Abcam), or α-tubulin (1:4,000, AC007, ABclonal). After primary incubation, appropriate horseradish peroxidase (HRP)-labeled secondary antibodies (1:10,000, ABclonal) were incubated with membranes for 1 h at room temperature. Signal revelation was performed using an enhanced chemiluminescence substrate (RM00021, ABclonal) detection system. Band intensities were quantified using the ImageJ analysis program.

### Immunofluorescence staining

The mice were anesthetized with 1% pentobarbital sodium and subsequently fixed *via* transcardial perfusion with 0.01 M phosphate-buffered saline (PBS) and ice-cold 4% PFA. After additional overnight fixation in 4% PFA at 4°C, brain tissues were infiltrated with 20% and 30% sucrose sequentially for 2 days and frozen in the Tissue-Tek OCT compound (Sakura Finetek). Brain tissues were cut into 40-μm-thick sections by a cryostat (Leica, CM1950), and the sections were stored in an ice-cold cryoprotective solution (30% glycerin, 30% propylene glycol, and 40% PBS) at −20°C. After being incubated in PBS with 0.3% Triton X-100 for 30 min and blocking buffer (10% normal goat serum, 3% BSA, and 0.3% Triton X-100, in PBS) for 1 h at room temperature, sections were incubated with primary antibodies in blocking buffer at 4°C overnight. After washing in PBS, sections were incubated with secondary antibodies for 2 h at room temperature, following additional washing in PBS. Finally, sections were mounted onto microscopy slides with a mounting medium (containing DAPI) (S2110, Beijing Solarbio Science & Technology, China) and subjected to image processing. Images were acquired using a virtual slide microscope (Olympus, SV120) or a confocal microscope (Carl Zeiss, LSM780). Images were analyzed with ImageJ and postprocessed using Adobe Photoshop CC. Cells were manually counted in images collected for each mouse.

To characterize the expression pattern of AAV-GFP or AAV-LEAP2 in the ARC, mice infected with AAV in the ARC for 4 weeks on a chow diet were perfused, and brain sections were prepared as above mentioned. Sections containing ARC were stained with NeuN for neuronal cells and glial fibrillary acidic protein (GFAP) for astrocytes. The primary antibodies were mouse anti-NeuN (1:500, ab104224, Abcam) and mouse anti-GFAP (1:200, A11864, ABclonal), and the secondary antibody was Alexa Fluor 594–conjugated goat anti-mouse IgG (1:250, SA00006-3, Proteintech).

The expression of c-Fos in POMC neurons after LEAP2 treatment was examined in POMC-Cre;Rosa-tdTomato mice. Specifically, male POMC-Cre;Rosa-tdTomato mice were handled for 3 days. On the fourth day, they were i.c.v. injected with aCSF or LEAP2 (10 nM in 2 μl) in the morning and kept food-restricted for 2 h before being sacrificed. The primary and the secondary antibodies were rabbit anti-c-Fos (1:500, ab214672, Abcam) and Alexa Fluor 488–conjugated goat anti-rabbit IgG (1:250, AS053, ABclonal), respectively.

To verify the specificity of GCaMP6m expression in POMC neurons, POMC immunostaining was performed in the sections containing ARC from mice infected with the Cre-dependent GCaMP6m protein. To determine whether hM4Di was selectively expressed in POMC neurons, POMC immunostaining was performed in the sections containing ARC from mice infected with the Cre-dependent hM4Di protein. The primary and secondary antibodies were rabbit anti-POMC (1:300, BM5411, BOSTER) and Alexa Fluor 594–conjugated goat anti-rabbit IgG (1:250, SA00006-4, Proteintech), respectively.

### Calcium imaging

Calcium imaging was conducted according to a previously described method (36, 37). GCaMP6m was selectively expressed in ARC POMC neurons through AAV-mediated, Cre-dependent expression (rAAV-EF1α-DIO-GCaMP6m, 200 nl/side) in adult POMC-Cre mice. Three to four weeks after stereotaxic injection of the virus, brain tissues were quickly dissected and placed in ice-cold aCSF cutting solution (in mM): 110 choline chloride, 2.5 KCl, 1.3 NaH_2_PO_4_, 25 NaHCO_3_, 20 glucose, 1.3 Na-ascorbate, 0.6 Na-pyruvate, 0.5 CaCl_2_, and 7 MgCl_2_. Brain tissues were sliced into 275-μm-thick sections on a vibratome (Leica, VT1000S) and transferred to 34°C oxygenated standard aCSF (125 NaCl, 2.5 KCl, 1.3 NaH_2_PO_4_, 25 NaHCO_3_, 10 glucose, 1.3 Na-ascorbate, 0.6 Na-pyruvate, 2 CaCl_2_, 1.3 MgCl_2_) for 30 min, and then moved to room temperature aCSF for additional 1 h. All solutions were saturated with a mixture of 95% O_2_/5% CO_2_ (vol/vol).

For Ca^2+^ imaging, sections were placed in a recording chamber superfused with oxygenated aCSF (2 ml/min) on the stage of an upright microscope (Olympus, BX51WI). Cells were imaged for GCaMP6m fluorescence using a 40× water immersion lens (Olympus, NA = 1.0). GCaMP6m fluorescence was recorded for 20 seconds before LEAP2 application to assess the general activity of POMC neurons. The sections were then treated with LEAP2 (100 nM, bath superfusion) for 5 min, and imaging was then performed. Images were collected at 10 frames per second using Image-Pro Plus software. Image stacks were processed using ImageJ, and GCaMP6m fluorescence was quantified using MATLAB R2020b (Mathworks, Natick, MA) with customized code and Cellsort 4.0 software (Thinkertech, Nanjing, China) for the soma of individual cells. After correction and denoising, the regions of interest were selected manually according to the fluorescence intensity. The average calcium signals in the first 1.5 seconds were considered the baseline and used as a reference (*F*_0_) to normalize the fluorescence signal (Δ*F/F*). The formula is as follows:


$$\Delta F/F=\frac{F_{signal}-F_{0}}{F_{0}}$$

*F*_signal_ represents the real-time calcium signal intensity of the target cells.

### Chemogenetic manipulation of POMC neurons and electrophysiology

To determine whether POMC neurons mediate the anorexigenic effect of LEAP2, AAV-DIO-hM4Di-EGFP was stereotaxically delivered into the ARC of male POMC-Cre (POMC-hM4Di) or WT (control) littermates (200 nl/side). Mice were further implanted with a cannula into the third ventricle for infusion of LEAP2 2 weeks after virus injection. After 1 week of recovery, 3 mg/kg CNO (HY-17366, MedChemExpress) or 0.9% saline was i.p. injected in POMC-hM4Di mice at 19:00. Food intake was monitored at 2, 4, and 12 h after i.p. injection. After a rest for 3 days, both control and POMC-hM4Di mice were subjected to CNO (3 mg/kg, i.p.) + aCSF (i.c.v.) or CNO (3 mg/kg, i.p.) + LEAP2 (10 nM, i.c.v.). Food intake was recorded 4 h after i.c.v. injection. To verify injection accuracy, GFP signals on ARC-containing brain slices were monitored under a fluorescent microscope.

To validate the chemogenetic effect in POMC-hM4Di mice, 2 weeks after AAV-DIO-hM4Di-EGFP virus infection, ARC-containing brain slices were prepared, and the effects of CNO (10 µM, bath superfusion) and CNO + LEAP2 (100 nM) on the resting membrane potential (RMP) and firing rate of GFP-labeled POMC neurons were recorded using similar protocols, as described previously (38). Briefly, brain tissues were sliced into 275-µm-thick sections with vibratome in ice-cold aCSF cutting solution and incubated at 34°C in oxygenated standard aCSF solution for 30 min, then placed in the recording chamber constantly perfused with aCSF at room temperature. Whole-cell patch-clamp recordings were performed in current-clamp mode, and membrane voltage was recorded from GFP-expressing POMC neurons using electrodes with 4–6 MΩ tip resistances. Pipette was tip-filled with solution composed of (in mM): 145 K-gluconate, 1 MgCl_2_, 10 HEPES, 1.1 EGTA, 2 Mg-ATP, 0.5 Na_2_-GTP, and 5 Na_2_-phosphocreatine (pH 7.3; 290 mOsm). MultiClamp 700B Amplifier (Molecular Devices, San Jose, CA, USA) and Axon pCLAMP 11.2 software (Molecular Devices, San Jose, CA, USA) were used to acquire and analyze data.

### Statistical analysis

All data were presented as mean ± standard error of the mean (SEM). As shown in the figure legends, data were analyzed using the two-tailed Student’s *t*-test and one- or two-way ANOVA in GraphPad Prism 8 software. *p* values below 0.05 were considered statistically significant.

## Results

### LEAP2 overexpression in the ARC results in hypophagia and reduces body weight gain in chow-fed mice

**Figure 1:**
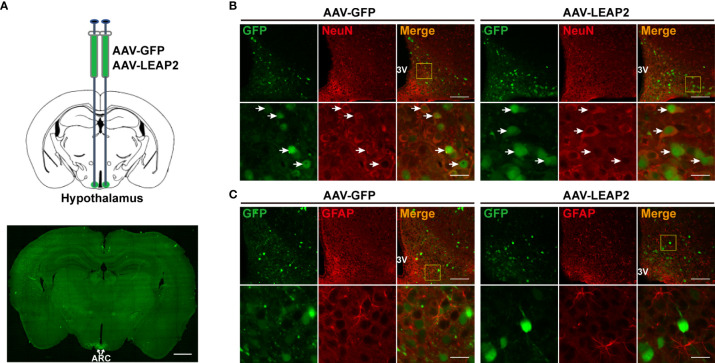
Overexpression of LEAP2 in the ARC neurons. **(A)** AAV-GFP or AAV-LEAP2 was injected into the ARC of adult male mice (top), and GFP was expressed in the ARC (bottom). Scale bar, 1 mm. **(B)** Representative images of GFP and NeuN immunostaining performed 4 weeks after the last injection of AAV-GFP or AAV-LEAP2. The GFP expression by AAV-GFP or AAV-LEAP2 was restricted within the neurons. 3V, third ventricle. Arrows indicate cells labeled with GFP and NeuN; 100 and 20 μm for the low- and high-magnification images, respectively. **(C)** Representative images of GFP and GFAP immunostaining performed 4 weeks after the last injection of AAV-GFP or AAV-LEAP2. 3V, third ventricle; 100 and 20 μm for the low- and high-magnification images, respectively.

**Figure 2:**
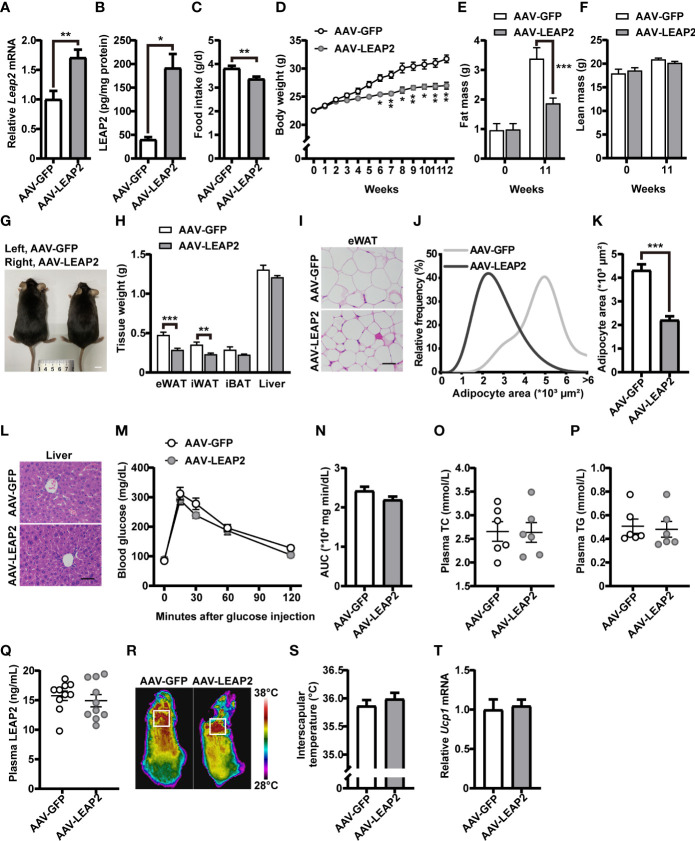
LEAP2 overexpression in the ARC of mice reduces food intake and body weight without altering metabolism on a chow diet. **(A)** Relative *Leap2* mRNA expression in the hypothalamus of mice injected with AAV-GFP or AAV-LEAP2 at 4 weeks after virus injection on a chow diet. *n* = 6/group, two-tailed Student’s *t*-test, *t* = 3.560, ^**^ *p* = 0.0052< 0.01. **(B)** Hypothalamic content of LEAP2 peptide in mice injected with AAV-GFP or AAV-LEAP2 at 4 weeks after virus injection on a chow diet. *n* = 4/group, two-tailed Student’s *t*-test, *t* = 5.019, ^*^ *p* = 0.0132< 0.05. **(C–F)** LEAP2 overexpression in the ARC reduced food intake **(C)**, weekly body weight **(D)**, and total fat mass **(E)**, without affecting total lean mass **(F)**, assessed in normal chow-fed mice during the 12 weeks after virus injection. *n* = 8/group, two-tailed Student’s *t*-test, *t* = 3.218, ^**^ *p* = 0.0062< 0.01 **(C)**. *n* = 8/group, two-way ANOVA with Bonferroni’s *post-hoc* test, *F (1*, 14) = 28.70, ^***^ *p* = 0.0001< 0.001 for virus **(D)**. *n* = 6–8/group, two-way ANOVA with Bonferroni’s *post hoc* test, *F*_(1, 24)_ = 7.577, ^*^ *p* = 0.0111< 0.05 for virus, ^***^ *p* = 0.0005< 0.001, 11 weeks **(E)**; *F*_(1, 24)_ = 0.01153, *p* = 0.9154 for virus **(F)**. **(G)** Representative photograph of mice fed a chow diet and injected with AAV-GFP or AAV-LEAP2 12 weeks after virus injection. Scale bars, 1 cm. *n* = 8/group. **(H)** Individual fat tissue and liver weights of mice fed a chow diet and injected with AAV-GFP or AAV-LEAP2 12 weeks after virus injection. *n* = 8/group, two-tailed Student’s *t*-test, *t* = 4.532, ^***^ *p* = 0.0005< 0.001 for eWAT; *t* = 3.461, ^**^ *p* = 0.0065< 0.01 for iWAT. **(I)** H&E-stained sections of eWAT from mice fed a chow diet and injected with AAV-GFP or AAV-LEAP2 at 12 weeks after virus injection. Scale bar, 50 μm. **(J, K)** Distribution of adipocyte area **(J)** and the average area **(K)** in eWAT. *n* = 5/group, two-tailed Student’s *t*-test, *t* = 7.111, ^***^ *p* = 0.0001< 0.001 **(K)**. **(L)** Representative images of liver histology with H&E staining in mice fed a chow diet and injected with AAV-GFP or AAV-LEAP2 at 12 weeks after virus injection. Scale bar, 50 μm. **(M, N)** No difference was observed in the glucose tolerance test (GTT) at week 11 after virus injection **(M)**. The corresponding area under the curve (AUC) of the GTT **(M)** was calculated **(N)**. *n* = 7/group, two-way ANOVA, *F*_(1, 12)_ = 2.837, *p* = 0.1180 **(M)**; two-tailed Student’s *t*-test, *p* > 0.05 **(N)**. **(O, P)** No difference was observed in fasting plasma TC **(O)** and TG **(P)** levels of the mice fed a chow diet for 12 weeks. *n* = 6/group, two-tailed Student’s *t*-test, *p* > 0.05. **(Q)** No difference was observed in fasting plasma LEAP2 levels of the mice fed a chow diet for 12 weeks. *n* = 10/group, two-tailed Student’s *t*-test, *p* > 0.05. **(R, S)** Representative infrared thermography of mice fed a chow diet at week 12 after virus injection **(R)**. Surface temperature in the interscapular BAT area represented by a square was quantified **(S)**. *n* = 8/group, two-tailed Student’s *t*-test, *p* > 0.05 **(S)**. **(T)** Relative *Ucp1* mRNA expression in BAT had no change in the AAV-LEAP2 group compared to the AAV-GFP group fed a chow diet for 12 weeks. *n* = 5/group, two-tailed Student’s *t*-test, *p* > 0.05. Data are presented as mean ± SEM. ^**^ *p*< 0.01; ^***^ *p*< 0.001.

To investigate the effects of chronic ARC LEAP2 administration on metabolic balance, AAV-GFP or AAV-LEAP2 was delivered stereotaxically to the bilateral ARC of standard chow-fed adult male WT mice. Four weeks after virus injection, GFP expression in the ARC was confirmed by fluorescence analysis (**Figure 1A**). All GFP^+^ cells were co-localized with NeuN (**Figure 1B**) but not GFAP (**Figure 1C**). Although LEAP2 in the CSF of the AAV-LEAP2 group was below the ELISA detection level (data not shown), the number of c-Fos-positive cells was increased specifically in the ARC (**Supplementary Figures S1A, B**), suggesting that LEAP2 overexpression stimulates neurons in the ARC. *Leap2* overexpression in the hypothalamus of AAV-LEAP2–injected mice was detected by qRT-PCR (**Figure 2A**). In addition, LEAP2 was produced and secreted by these transduced hypothalamic cells (**Figure 2B**). We also analyzed spontaneous locomotion activity, anxiety, and depression-like behaviors of mice overexpressing LEAP2 in ARC in the open field, elevated plus maze, tail suspension, and forced swimming tests. No gross behavioral changes were observed (**Supplementary Figures S2A–D**). We then evaluated the effects of LEAP2 on metabolic phenotypes.

Under a chow diet, exogenous expression of LEAP2 in the ARC by AAV-LEAP2 significantly reduced food intake (**Figure 2C**) and body weight (**Figure 2D**), attenuated fat mass (**Figure 2E**) but not lean mass (**Figure 2F**), and showed smaller body size up to 20 weeks of age (**Figure 2G**). Moreover, the wet weight and histology of tissues important for regulating nutrient metabolism were examined. The weight of the eWAT and iWAT were all remarkably decreased in mice treated with AAV-LEAP2, without significant changes in the iBAT (**Figure 2H**). Further histology analysis showed a significant decrease in average adipocyte size in the eWAT of AAV-LEAP2–treated mice (**Figures 2I–K**) and no significant difference in liver histology in these two groups (**Figure 2L**), consistent with the reduced and similar wet weight of eWAT and liver, respectively (**Figure 2H**). We also observed glucose tolerance (**Figures 2M, N**), plasma TC (**Figure 2O**), and TG (**Figure 2P**) levels were unaffected, and there were no changes in the fasting plasma LEAP2 levels in AAV-LEAP2–injected mice (**Figure 2Q**). Concerning energy metabolism, we assessed the interscapular temperature and the level of mRNA encoding the mitochondrial proton carrier uncoupling protein 1 (UCP1) in the BAT of mice, a known marker for enhanced thermogenesis (39, 40). Mice injected with AAV-LEAP2 into the ARC did not significantly affect interscapular temperature (**Figures 2R, S**) or *Ucp1* expression in BAT (**Figure 2T**). These results indicate that LEAP2 overexpression in the ARC by AAV-LEAP2 injection suppresses appetite, accompanied by lower body weight and changes in body composition in chow-fed mice. This leaner phenotype results from decreased food intake rather than alteration of energy metabolism.

### LEAP2 overexpression in the ARC overrides the ghrelin action of mice exposed to a chow diet

**Figure 3:**
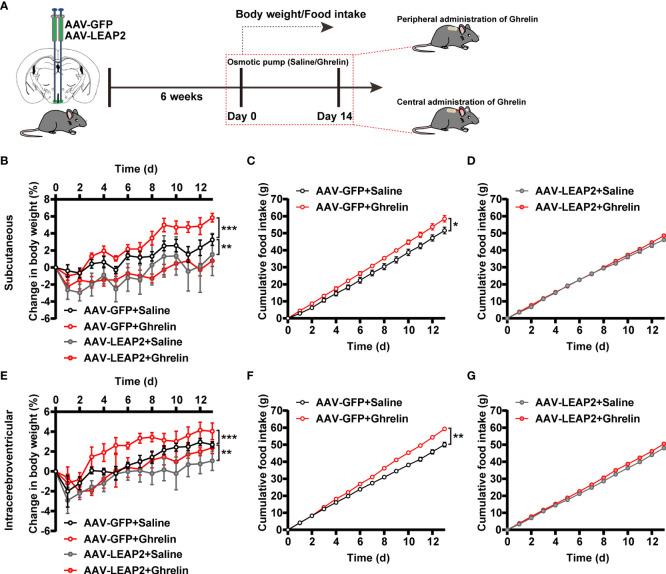
LEAP2 overexpression in the ARC blunts the increase in body weight gain and food intake associated with chronic ghrelin administration on a chow diet. **(A)** Schematic diagram showing chronic central and peripheral administration of acyl-ghrelin by implanted osmotic pumps in virus-transduced AAV-GFP or AAV-LEAP2 in the ARC of WT mice. Body weight and food intake were measured daily. **(B–D)** Changes in body weight **(B)** and cumulative food intake **(C, D)** in virus-transduced AAV-GFP or AAV-LEAP2 WT mice following continuous subcutaneous administration of saline or ghrelin by osmotic pumps for 2 weeks. *n* = 6/group, two-way ANOVA with Bonferroni’s *post-hoc* test, *F*_(3, 20)_ = 3.183, ^*^ *p* = 0.0462< 0.05 for treatment; ^**^ *p* = 0.0058< 0.01 for AAV-GFP + saline vs. AAV-LEAP2 + saline, ^***^ *p* = 0.0005< 0.001 for AAV-GFP + saline vs. AAV-GFP + ghrelin, *p* > 0.05 for AAV-LEAP2 + saline vs. AAV-LEAP2 + ghrelin **(B)**. *n* = 6/group, two-way ANOVA, *F*_(1, 10)_ = 6.573, ^*^ *p* = 0.0282< 0.05 **(C)**. *n* = 6/group, two-way ANOVA, *F*_(1, 10)_ = 0.9124, *p* = 0.3620 > 0.05 **(D)**. **(E–G)** Changes in body weight **(E)** and cumulative food intake **(F, G)** in virus-transduced AAV-GFP or AAV-LEAP2 WT mice following chronic administration of saline or ghrelin into the lateral ventricle by osmotic pumps for 2 weeks. *n* = 6/group, two-way ANOVA with Bonferroni’s *post hoc* test, *F*_(3, 20)_ = 4.411, * *p* = 0.0155< 0.05 for treatment; ** *p* = 0.0018< 0.01 for AAV-GFP + saline vs. AAV-LEAP2 + saline, ^***^ *p* = 0.0007< 0.001 for AAV-GFP + saline vs. AAV-GFP + ghrelin, *p* > 0.05 for AAV-LEAP2 + saline vs. AAV-LEAP2 + ghrelin **(E)**. *n* = 6/group, two-way ANOVA, *F*_(1, 10)_ = 20.93, ^**^ *p* = 0.001< 0.01 **(F)**. *n* = 6/group, two-way ANOVA, *F*_(1, 10)_ = 4.887, *p* = 0.0515 > 0.05 **(G)**. Data are presented as mean ± SEM. ^*^ *p*< 0.05; ^**^ *p*< 0.01; ^***^ *p*< 0.001.

Given the concentrated GHSR expression in the ARC (41) and that LEAP2 is thought to inhibit ghrelin action through inverse effects on GHSR activity (2), we asked whether ghrelin attenuates the effects of LEAP2 overexpression in the ARC on body weight and food intake. Six weeks after the injection of AAV-GFP or AAV-LEAP2 into the ARC, we administered acyl-ghrelin or saline chronically to these mice *via* subcutaneous or intracerebroventricular osmotic mini-pumps for 2 weeks (**Figure 3A**). As anticipated, both central and peripheral continuous infusions of acyl-ghrelin dramatically elevated body weight gain, and more food was consumed relative to the vehicle in AAV-GFP–treated control mice. However, acyl-ghrelin administration had no effect on AAV-LEAP2–induced less body-weight gain and food intake (**Figures 3B–G**). These data suggest that the effects of 14-day acyl-ghrelin infusion were blunted in the AAV-LEAP2–injected mice.

### LEAP2 overexpression in the ARC ameliorates high-fat diet–induced obesity and associated comorbidities

**Figure 4:**
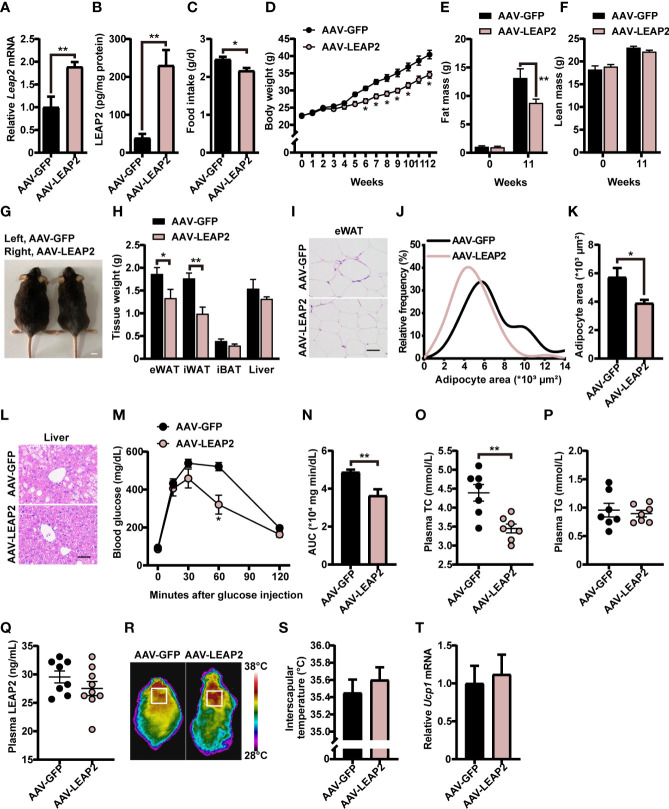
LEAP2 overexpression in the ARC reduces food intake, body weight, and adiposity and improves hepatic steatosis, glucose tolerance, and blood lipids in high-fat diet–fed mice. **(A)** Relative *Leap2* mRNA expression in the hypothalamus of mice injected with AAV-GFP or AAV-LEAP2 at 4 weeks after virus injection on a HFD. *n* = 6/group, two-tailed Student’s *t*-test, *t* = 3.476, ^**^ *p* = 0.0060< 0.01. **(B)** Hypothalamic content of LEAP2 peptide in mice injected with AAV-GFP or AAV-LEAP2 at 4 weeks after virus injection on a HFD. *n* = 4/group, two-tailed Student’s *t*-test, *t* = 4.573, ** *p* = 0.0038< 0.01. **(C–F)** LEAP2 overexpression in the ARC reduced food intake **(C)**, weekly body weight **(D)**, and total fat mass **(E)**, without affecting total lean mass **(F)**, assessed in HFD-fed mice during the 12 weeks after virus injection. *n* = 8/group, two-tailed Student’s *t*-test, *t* = 2.979, ^*^ *p* = 0.01< 0.05 **(C)**. *n* = 8/group, two-way ANOVA with Bonferroni’s *post-hoc* test, *F*_(1, 14)_ = 13.90, ^**^ *p* = 0.0022< 0.01 for virus **(D)**. *n* = 6–8/group, two-way ANOVA with Bonferroni’s *post-hoc* test, *F*_(1, 24)_ = 4.817, * *p* = 0.0381< 0.05 for virus, ^**^ *p* = 0.0061< 0.01, 11 weeks **(E)**; *F*_(1, 24)_ = 0.05832, *p* = 0.8112 for virus **(F)**. **(G)** Representative photograph of mice fed a HFD and injected with AAV-GFP or AAV-LEAP2 at 12 weeks after virus injection. Scale bars, 1 cm. *n* = 8/group. **(H)** Weight of individual fat tissues and liver of mice fed a HFD and injected with AAV-GFP or AAV-LEAP2 at 12 weeks after virus injection. *n* = 6/group, two-tailed Student’s *t*-test, *t* = 2.258, ^*^ *p* = 0.0475< 0.05 for eWAT; *t* = 4.125, ^**^ *p* = 0.0021< 0.01 for iWAT. **(I)** H&E-stained sections of eWAT from mice fed a HFD and injected with AAV-GFP or AAV-LEAP2 at 12 weeks after virus injection. Scale bar, 50 μm. **(J, K)** Distribution of adipocyte area **(J)** and the average area **(K)** in eWAT. *n* = 5/group, two-tailed Student’s *t*-test, *t* = 2.646, ^*^ *p* = 0.0294< 0.05 **(K)**. **(L)** Representative images of liver histology with H&E staining in mice fed a HFD and injected with AAV-GFP or AAV-LEAP2 at 12 weeks after virus injection. Scale bar, 50 μm. **(M, N)** Mice injected with AAV-LEAP2 utilized glucose more efficiently compared with control mice fed a HFD at week 11 after virus injection **(M)**. The corresponding AUC of the GTT **(M)** was calculated **(N)**. *n* = 6/group, two-way ANOVA with Bonferroni’s *post-hoc* test, *F*_(1, 10)_ = 9.367, ^*^ *p* = 0.0120< 0.05 for virus, ^*^ *p* = 0.0406< 0.05, 60 min **(M)**; two-tailed Student’s *t*-test, *t* = 3.458, ^**^ *p* = 0.0061< 0.01 **(N)**. **(O, P)** Mice injected with AAV-LEAP2 had lower fasting plasma TC levels compared with control mice fed a HFD for 12 weeks **(O)**. No difference was observed in the fasting plasma TG levels of these mice **(P)**. *n* = 7/group, two-tailed Student’s *t*-test, *t* = 3.790, ^**^ *p* = 0.0026< 0.01 **(O)**, *p* > 0.05 **(P)**. **(Q)** No difference was observed in the fasting plasma LEAP2 levels of the mice fed a HFD for 12 weeks. *n* = 8–9/group, two-tailed Student’s *t*-test, *p* > 0.05. **(R, S)** Representative infrared thermography of mice fed a HFD at week 12 after virus injection **(R)**. Surface temperature in the interscapular BAT area represented by a square was quantified **(S)**. *n* = 8/group, two-tailed Student’s *t*-test, *p* > 0.05 **(S)**. **(T)** Relative *Ucp1* mRNA expression in BAT had no change in the AAV-LEAP2 group compared to the AAV-GFP group fed a HFD for 12 weeks. *n* = 5/group, two-tailed Student’s *t*-test, *p* > 0.05. Data are presented as mean ± SEM. ^*^ *p*< 0.05; ^**^ *p*< 0.01.

We next explored whether these effects of overexpression of LEAP2 in the ARC are conserved during the HFD challenge. A separate cohort of adult male WT AAV-GFP– or AAV-LEAP2–injected mice were fed HFD for 12 weeks to induce obesity after surgical recovery. After confirming the efficiency of overexpression (**Figures 4A, B**), we found that mice overexpressing LEAP2 in the ARC consumed considerably less HFD and gained less weight than the control group (**Figures 4C, D, G**), as observed under conditions of chow diet feeding. Thus, the anorexigenic effect of LEAP2 overexpression in the ARC might be independent of the caloric level of the diet. A significant decrease in fat mass (**Figure 4E**) but not lean mass (**Figure 4F**) was observed in AAV-LEAP2-treated mice compared with AAV-GFP controls, attributed mainly to the reduced WAT mass, revealed by decreased eWAT and iWAT and unaffected iBAT mass (**Figure 4H**). Consistently, the average adipocyte size of eWAT was significantly smaller in mice injected with AAV-LEAP2 into the ARC (**Figures 4I–K**). Additionally, HFD-fed mice subjected to AAV-LEAP2 exhibited less hepatosteatosis (**Figure 4L**). Unlike in chow-fed animals, the intraperitoneal glucose tolerance test (IPGTT) revealed that AAV-LEAP2–injected mice were less glucose-intolerant during HFD feeding, indicating improved glucose utilization (**Figures 4M, N**). Moreover, LEAP2 overexpression in the ARC reduced plasma TC levels but not TG, suggesting improved dysregulation of lipid metabolism in HFD-fed mice (**Figures 4O, P**). As in the chow-fed animals, plasma LEAP2 levels in the AAV-LEAP2 group were unaltered compared with the control group in HFD-fed mice (**Figure 4Q**). Finally, the interscapular temperature of AAV-LEAP2–treated mice was similar to the AAV-GFP controls (**Figures 4R, S**). Consistently, no difference in *Ucp1* expression in BAT was observed (**Figure 4T**). Hence, these data showed that the central overexpression of LEAP2 in the ARC protected mice against HFD-induced obesity and related metabolic disorders. Collectively, LEAP2 overexpression in the ARC reduces food intake and body weight, both under chow-fed and obesogenic conditions, indicating that the ARC is a LEAP2-responsive area of the hypothalamus.

### LEAP2 overexpression in the ARC promotes POMC expression

**Figure 5:**
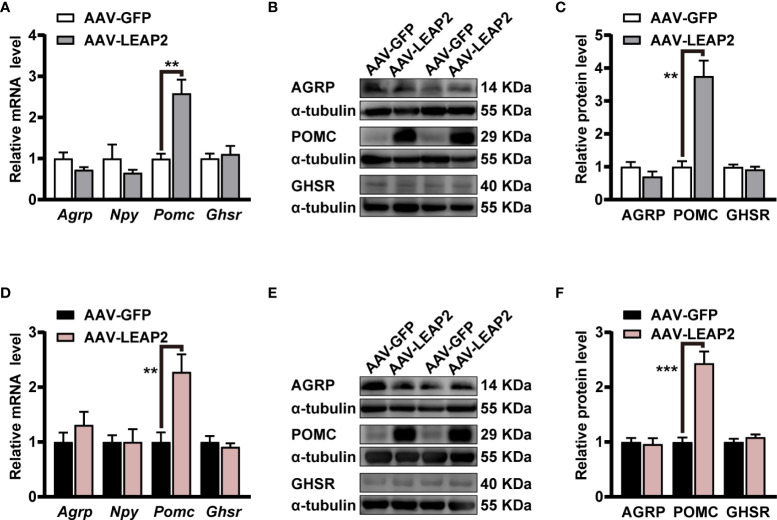
LEAP2 overexpression in the ARC increases the hypothalamic POMC expression but no change of AGRP, NPY, and GHSR in mice fed chow or a high-fat diet. **(A)** Relative mRNA levels of *Agrp*, *Npy*, *Pomc*, and *Ghsr* in the hypothalamus of mice fed a chow diet and injected with AAV-GFP or AAV-LEAP2 for 12 weeks. *n* = 6/group, two-tailed Student’s *t*-test, *t* = 4.481, ^**^ *p* = 0.0037< 0.01 for *Pomc*. **(B, C)** Representative immunoblot images of proteins **(B)** and densitometric quantification **(C)** for AGRP, POMC, and GHSR in the hypothalamus of mice fed a chow diet after virus injection for 12 weeks. α-Tubulin was used as the loading control. *n* = 4–6/group, two-tailed Student’s *t*-test, *t* = 5.490, ^**^ *p* = 0.0014< 0.01 for POMC **(C)**. **(D)** Relative mRNA levels of *Agrp*, *Npy*, *Pomc*, and *Ghsr* in the hypothalamus of mice fed a HFD and injected with AAV-GFP or AAV-LEAP2 for 12 weeks. *n* = 6/group, two-tailed Student’s *t*-test, *t* = 3.513, ^**^ *p* = 0.0056< 0.01 for *Pomc*. **(E, F)** Representative immunoblot images of proteins **(E)** and densitometric quantification **(F)** for AGRP, POMC, and GHSR in the hypothalamus of mice fed a HFD after virus injection for 12 weeks. α-Tubulin was used as the loading control. *n* = 4–6/group, two-tailed Student’s *t*-test, *t* = 6.281, ^***^ *p* = 0.0002< 0.001 for POMC **(F)**. Data are presented as mean ± SEM. ^**^ *p*< 0.01; ^***^ *p*< 0.001.

As our results showed changes in food intake and body weight by the overexpression of LEAP2 in the ARC, we further evaluated the expression of some neuropeptides and GHSR in the hypothalamus. Interestingly, qRT-PCR results demonstrated that mRNA of anorexigenic *Pomc* in the hypothalamus was significantly elevated by AAV-LEAP2 treatment in mice fed chow or HFD, whereas that of orexigenic *Agrp* and *Npy* were unaltered (**Figures 5A, D**). In addition, the *Ghsr* level in the hypothalamus was not changed in AAV-LEAP2–injected mice under chow or HFD conditions (**Figures 5A, D**). Consistently, Western blot analysis further confirmed that AAV-LEAP2 injection into the ARC stimulated hypothalamic POMC protein expression and did not change AGRP and GHSR protein expression in mice fed chow or HFD (**Figures 5B, C, E, F**). Taken together, these results suggest that LEAP2 overexpression in the ARC increases POMC expression and that this effect is implicated in the anorexigenic effect of AAV-LEAP2. Hence, in chow- and HFD-fed mice, increased production of POMC at least partially accounts for the effects of overexpression of LEAP2 in the ARC.

### Central administration of LEAP2 suppresses appetite and increases the POMC neuronal activity in ARC

**Figure 6:**
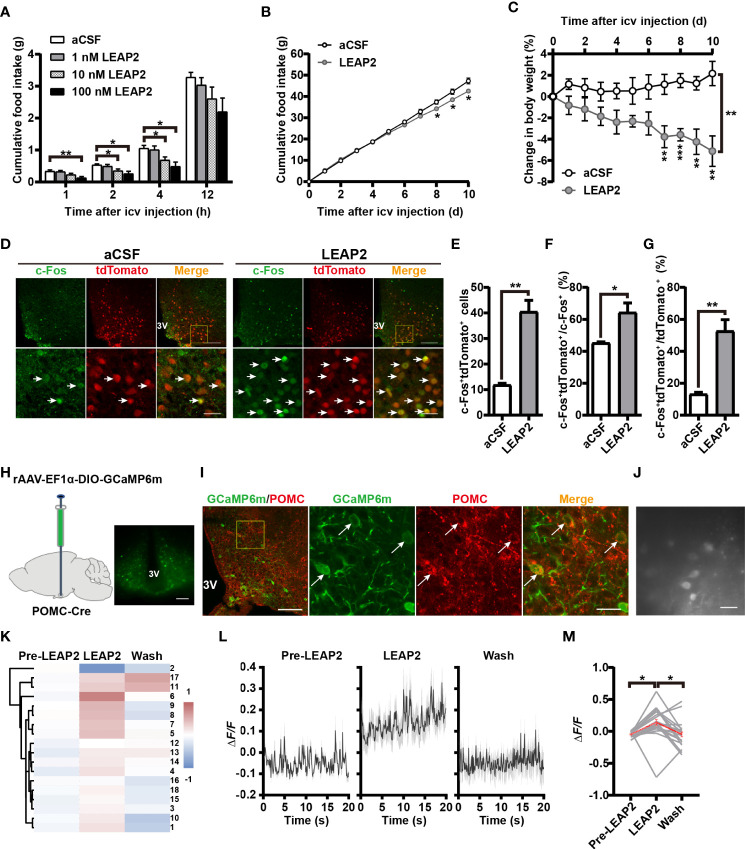
LEAP2 decreases food intake and body weight and increases POMC neuronal activity in ARC. **(A)** Cumulative food intake during the dark cycle (12 h) of WT mice after an acute single i.c.v.-administered aCSF or LEAP2. *n* = 16–18/group, two-way ANOVA with Bonferroni’s *post-hoc* test, *F*_(3, 66)_ = 4.443, ^**^ *p* = 0.0066< 0.01 for treatment, ^**^ *p* = 0.0035< 0.01 for aCSF vs. 100 nM LEAP2, 1 h; ^*^ *p* = 0.0433< 0.05 for aCSF vs. 10 nM LEAP2, ^*^ *p* = 0.0150< 0.05 for aCSF vs. 100 nM LEAP2, 2 h; ^*^ *p* = 0.0489< 0.05 for aCSF vs. 10 nM LEAP2, ^*^ *p* = 0.0107< 0.05 for aCSF vs. 100 nM LEAP2, 4 h. **(B, C)** Cumulative food intake **(B)** and change in body weight **(C)** of WT mice during i.c.v. administration of aCSF or LEAP2 (10 nM) once each day for 10 days. *n* = 6/group, two-tailed Student’s *t*-test, *t* = 2.782, ^*^ *p* = 0.0194< 0.05, 8 days; *t* = 2.623, ^*^ *p* = 0.0255< 0.05, 9 days; *t* = 3.102, ^*^ *p* = 0.0112< 0.05, 10 days **(B)**. Two-way ANOVA with Bonferroni’s *post-hoc* test, *F*_(1, 10)_ = 20.54, ^**^ *p* = 0.0011< 0.01 for treatment **(C)**. **(D)** Staining of c-Fos in the ARC of POMC-Cre;Rosa-tdTomato mice treated with a single i.c.v. injection of either aCSF or LEAP2 (10 nM). 3V, third ventricle. Arrows indicate c-Fos and tdTomato double-positive cells; 100 and 25 μm for the low- and high-magnification images, respectively. **(E)** The number of c-Fos^+^ and tdTomato^+^ cells in the ARC of mice administered aCSF or LEAP2. *n* = 6/group, two-tailed Student’s *t*-test, *t* = 6.408, ^**^ *p* = 0.0012< 0.01. **(F, G)** The ratio of c-Fos and tdTomato double-positive cells to c-Fos-positive **(F)** and tdTomato-positive **(G)** cells. n = 6/group, two-tailed Student’s t-test, *t* = 3.230, * *p* = 0.0222< 0.05 **(F)**; *t* = 5.495, ** *p* = 0.0023< 0.01 **(G)**. **(H)** Bilateral injection of rAAV-EF1α-DIO-GCaMP6m into the ARC of POMC-Cre mice. Representative coronal section image showing GCaMP6m expression in the ARC of POMC-Cre mice. Scale bar, 200 μm. **(I)** Representative photographs showing the expression of POMC in GCaMP6m-positive neurons in the ARC of POMC-Cre mice. 3V, third ventricle. Arrows indicate GCaMP6m and POMC double-positive cells. 100 and 25 μm for the low and high-magnification images, respectively. **(J)** Example field of view from the microscope showing POMC cells. Scale bar, 50 μm. **(K)** Hierarchical cluster analysis of recorded neurons and heatmap of average Δ*F/F* of activity in the three stages, including pre-LEAP2, LEAP2 (100 nM) application, and washing out. **(L)** Response profiles (mean ± SEM) of POMC neuron clusters showing average activity traces aligned to pre-LEAP2, LEAP2 (100 nM) application, and washing out. **(M)** Average Δ*F/F* of calcium signals of all recorded POMC neurons in the three stages. Gray lines refer to individual neurons; the red line reflects the mean value. *n* = 18 neurons from four mice, one-way ANOVA with Bonferroni’s *post-hoc* test, *F*_(2, 51)_ = 5.257, ^**^ *p* = 0.0084< 0.01; ^*^ *p* = 0.0271< 0.05 for Pre-LEAP2 vs. LEAP2, ^*^ *p* = 0.0385< 0.05 for LEAP2 vs. wash. Data are presented as mean ± SEM. ^*^ *p*< 0.05; ^**^ *p*< 0.01.

In light of the above findings, we further explored the feeding-inhibition effect of LEAP2 through a central mechanism. To examine the acute effect of i.c.v.-administered LEAP2 peptide on food consumption, we implanted a guide cannula into the third ventricle of chow-fed mice. After recovery, we injected control aCSF or the full-length LEAP2 ranging from 1 to 100 nM into the brain immediately before the dark cycle. LEAP2 injection at 10- and 100-nM doses significantly suppressed the animals’ appetites at 2 and 4 h postinjection over a 12-h observation period compared with the aCSF group (**Figure 6A**). To understand chronic responses, we treated chow-fed mice with daily i.c.v. 10 nM LEAP2 or aCSF for 10 days. In addition to the expected hypophagia (**Figure 6B**), it is notable that these mice lost weight (**Figure 6C**). Therefore, consistent with the effects of AAV-LEAP2 in the ARC, prolonged hypophagia and sustained abatement of body weight gain were noted after directly introducing LEAP2 into the brain *via* i.c.v. injection.

Based on the observations that central administration of LEAP2 (AAV-LEAP2 injection into the ARC and LEAP2 peptide i.c.v. injection) induced an anorexigenic effect, we postulated that LEAP2 has a central anorexigenic mechanism. As POMC levels were elevated in response to LEAP2 overexpression in the ARC, we asked whether LEAP2 signals regulate POMC neuronal activity. POMC-Cre;Rosa-tdTomato reporter mice that express tdTomato specifically in POMC neurons were generated, and 90.4% of the tdTomato^+^ cells expressed POMC (**Supplementary Figures S3A, B**). c-Fos immunofluorescence in POMC-Cre;Rosa-tdTomato mice revealed that LEAP2 increased c-Fos expression in POMC neurons in the ARC after i.c.v. administered LEAP2 (10 nM) (**Figure 6D**), which was evidenced by an increased total number and proportion of c-Fos and tdTomato double-positive cells (**Figures 6E–G** **)**, indicating that LEAP2 might activate ARC POMC neurons.

To support this point of view, we used calcium imaging to examine the effect of LEAP2 on the activity of ARC POMC neurons. Four weeks after bilateral stereotactic injection of rAAV-EF1α-DIO-GCaMP6m into the ARC of POMC-Cre mice (**Figure 6H**), GCaMP6m expression in POMC neurons was confirmed using POMC immunostaining (**Figure 6I**). Acute brain slices were then prepared, and changes in GCaMP6m fluorescence were monitored using microscopy (**Figure 6J**). LEAP2 (100 nM) application to ARC-containing sections revealed a robust increase in Ca^2+^ concentration in POMC neurons relative to the pre-LEAP2 stage (**Figures 6K–M** **)**, indicating that LEAP2 can increase POMC neuronal activity.

### POMC neurons mediate the anorexigenic effect of LEAP2

**Figure 7:**
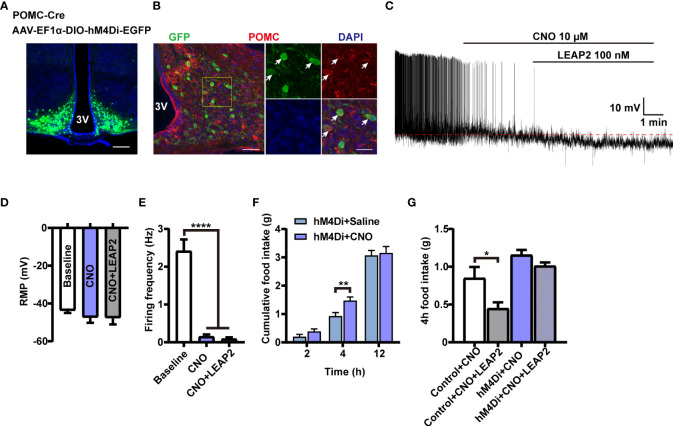
POMC neurons mediate the anorexigenic effect of LEAP2. **(A)** Bilateral injection of AAV-EF1α-DIO-hM4Di-EGFP into the ARC of POMC-Cre mice. Representative image of AAV-hM4Di-EGFP injection sites in the ARC. Scale bar, 200 μm. **(B)** Representative photographs showing the expression of POMC in hM4Di-positive neurons in the ARC of POMC-Cre mice. 3V, third ventricle. Arrows indicate hM4Di-EGFP and POMC double-positive cells; 100 and 25 μm for the low- and high-magnification images, respectively. **(C)** Representative trace of an electrophysiological response to CNO (10 μM, bath) and LEAP2 (100 nM) in ARC POMC neurons infected with AAV-hM4Di-EGFP. **(D, E)** Resting membrane potential (RMP) **(D)** and firing frequency **(E)**. *n* = 8 neurons from four mice, one-way ANOVA with Bonferroni’s *post-hoc* test, *F*_(2, 21)_ = 0.6111, *p* = 0.5521 **(D)**; *F*_(2, 21)_ = 52.55, ^****^ *p*< 0.0001 **(E)**. **(F)** Dark-cycle food intake measured in POMC-Cre mice infected with AAV-hM4Di-EGFP in the ARC treated with a single i.p. injection of either saline or CNO (3 mg/kg). *n* = 7/group, two-tailed Student’s *t*-test, *t* = 3.077, ^**^ *p* = 0.0096< 0.01 for hM4Di + saline vs. hM4Di + CNO, 4 h. **(G)** Effects of CNO (3 mg/kg, i.p.) co-injected with aCSF or LEAP2 (10 nM, i.c.v.) on dark-cycle food intake measured in WT or POMC-Cre mice infected with AAV-hM4Di-EGFP in the ARC. *n* = 8/group, one-way ANOVA with Bonferroni’s *post-hoc* test, *F*_(3, 28)_ = 10.44, ^****^ *p*< 0.0001; ^*^ *p* = 0.0332< 0.05 for control + CNO vs. control + CNO + LEAP2. Data are presented as mean ± SEM. ^*^ *p*< 0.05; ^**^ *p*< 0.01; ^****^ *p*< 0.0001.

To investigate whether the activation of POMC neurons mediates the anorexigenic effect of LEAP2 on food intake, we used chemogenetic technology to specifically silence POMC neurons. We generated POMC-hM4Di mice by stereotaxically delivering AAV-EF1α-DIO-hM4Di-EGFP into the ARC of POMC-Cre mice (**Figures 7A, B**). POMC neurons’ inhibition efficacy was confirmed by whole-cell recording on *ex vivo* slices. The CNO-driven activation of hM4Di decreased the firing frequencies of recorded POMC neurons while having little effect on the RMP, and LEAP2 (100 nM) cannot induce activation of POMC neurons in this situation (**Figures 7C–E**). CNO-mediated inhibition of POMC neurons increased dark-phase food intake (**Figure 7F**), and inhibition of POMC neurons significantly blocked acute LEAP2-induced feeding suppression (**Figure 7G**), demonstrating a mediating role of POMC neurons in the anorexigenic effect of LEAP2. A previous study showed that postnatal ablation of POMC neurons decreased food intake and led to enhanced anxiety-like behavior (42). Here, we analyzed the effects of CNO (3 mg/kg, i.p.) co-injected with aCSF or LEAP2 (10 nM, i.c.v.) on anxiety-like behaviors in these mice. We did not observe any significant changes in the open field test and elevated plus maze test (**Supplementary Figures S4A, B**).

## Discussion

The current study demonstrates the efficacy of central LEAP2 delivery in decreasing food intake and body weight *via* the activation of ARC POMC neurons. Firstly, long-term elevated expression of LEAP2 in the ARC reduces food intake, body weight, and adiposity with concomitantly improved hepatic steatosis, glucose tolerance, and blood lipids in mice exposed to HFD. Even on the standard chow diet, mice treated with AAV-LEAP2 in the ARC have less food intake and body weight. Since these mice did not exhibit increased energy expenditure, their lean phenotype is likely due to the anorexigenic effect of LEAP2. Notably, AAV-LEAP2–injected mice are insensitive to the effects of chronic acyl-ghrelin treatment on body weight and food intake. LEAP2 overexpression in the ARC does not affect AGRP and NPY expression but increases POMC expression in the hypothalamus. Furthermore, we show that the central administration of LEAP2 peptide is sufficient to induce acute POMC neuronal activation, lower food intake, and body weight, and ARC POMC neurons are critical mediators of the anorexigenic effect of LEAP2.

Several studies have examined the physiology of LEAP2 concerning the ghrelin system and metabolism (2, 8, 12–14), but its overall function is not well understood. It is uncertain whether LEAP2 treatment alone reduces food intake. Peripherally administered LEAP2 was originally reported to diminish 2-h food intake in mice compared to vehicle-treated mice (2). Subsequent experiments showed that i.c.v. administration of an N-terminal LEAP2 fragment decreased HFD eating during a binge-like eating protocol (16). Also, central LEAP2 treatment reduced food intake in mice fed a chow diet *ad libitum* (13). However, this effect could not be confirmed by others (5, 8, 14). Here, we took advantage of AAV vectors to overexpress LEAP2 in the ARC of adult mice, and its overexpression was confirmed by qRT-PCR and ELISA. Local overexpression of LEAP2 in the ARC would not influence animal locomotor activity and would induce mood disorders. However, these mice all exhibited hypophagia, reduced body weight, and adiposity in lean mice fed chow and HFD. In mice rendered obese by HFD, LEAP2 overexpression in the ARC also reduced hepatic steatosis, improved glucose tolerance, and decreased plasma total cholesterol level. Of note, LEAP2 overexpression in the ARC did not affect plasma levels of LEAP2 both in chow diet- and HFD-fed mice. The reason underlying this observation is unknown. It points out that LEAP2 sensing in the brain cannot drive LEAP2 production and release into the plasma from the liver or small intestine. Additionally, chronic overexpression of LEAP2 in the ARC did not result in remarkable differences in iBAT mass, interscapular temperature, or mRNA level of *Ucp1* in BAT, implying that the ARC-targeted LEAP2 overexpression did not induce peripheral BAT development or thermogenesis. Thus, these results indicate that central administration of AAV-LEAP2 in the ARC does not alter energy metabolism but rather reduces food intake, thereby lowering body weight and is responsible for the resistance to diet-induced obesity and related disorders. Chronic administration of ghrelin promotes weight gain in rodents (43), as well as increases food intake in humans (44). We found that centrally and peripherally administered acyl-ghrelin has no effect on body weight and food intake in mice transduced with AAV-LEAP2 in the ARC. LEAP2 can antagonize the effects of ghrelin on feeding, which supports the previous findings.

To explore the potential central molecular mechanisms, we investigated whether LEAP2 signaling regulates the expression of hypothalamic neuropeptides in the ARC. AAV-LEAP2 treatment induced dramatic upregulation of POMC expression in the hypothalamus but did not affect the expression of AGRP and NPY in lean mice and mice rendered obese by HFD. The anorexigenic peptide POMC is a crucial component of the melanocortin system, which involves the systemic and neural pathways that control energy balance (45–47). In early studies of POMC KO mice, the animals were diagnosed as hyperphagic or obese (48). Recent studies using optogenetic and chemogenetic stimulation of POMC neurons have shown decreased food intake (49, 50). Changes in hypothalamic POMC expression, but not AGRP and NPY expression, might account for the effects of LEAP2 overexpression in the ARC. POMC neurons suppress appetite by releasing the active MC4R agonist, α-melanocyte-stimulating hormone (α-MSH) (21, 46). It is plausible that POMC signaling, including active α-MSH and/or MC4R, mediates the effects of LEAP2 on food intake and body weight, but this requires further investigation. One report found that LEAP2 did not suppress NPY-induced feeding, prompting the authors of that study to conclude that “it suggests that NPY is not a target of LEAP2” (8). In a separate study, the powerful orexigenic effect of NPY was partially inhibited by exogenous LEAP2 administration in contrast to the expected complete suppression (13). Similarly, vertical sleeve gastrectomy in obese mice decreased body weight gain and adiposity and increased energy expenditure, demonstrating no change in the amount of AGRP/NPY-expressing neurons but an increase in the POMC-expressing neuron population (51). Although the present results suggest that LEAP2 does not regulate AGRP and NPY expression, we cannot rule out the possibility that alternative intracellular signaling pathways were activated in AGRP/NPY neurons and that these neuropeptides were involved in the process. Indeed, administration of LEAP2 peptide onto arcuate neurons led to hyperpolarization of NPY-positive neurons *in vitro* (9); administration of LEAP2 (1–12) partially abrogated the activation of ARC^AGRP/NPY^ neurons induced by food deprivation (52), suggesting LEAP2 may also interfere with orexigenic pathways. In addition, the possibility of other compensatory mechanisms in AGRP/NPY neurons or other neuropeptide systems cannot be excluded. Further studies are needed to explore this.

As a ghrelin and LEAP2 receptor, GHSR is widely distributed in the hypothalamus (53). In addition to neuropeptides, we examined the expression of GHSR. Interestingly, we did not detect any significant difference in this protein in the hypothalamus between the AAV-LEAP2 and the control AAV-injected mice under chow or HFD conditions. The questions raised in the present work are (1) whether or not LEAP2 can affect metabolic function independent of GHSR and (2) whether a new LEAP2 receptor might exist in these neurons. Future studies should clarify the precise role of GHSR activity in the neurobiology of LEAP2-mediated metabolic regulation.

Subsequently, we further confirmed LEAP2-mediated feeding inhibition through a central mechanism and explored its effect on hypothalamus ARC POMC neuronal activity. We i.c.v. injected WT mice with the full-length LEAP2 at different concentrations and monitored food intake in the dark cycle. Acute i.c.v. administration of LEAP2 dose-dependently decreased food intake relative to aCSF controls. Additionally, chronic i.c.v. infusion of LEAP2 for 10 days decreased cumulative food intake and body weight in mice fed a chow diet. Moreover, using POMC-Cre;tdTomato mice and immunofluorescence, we showed that LEAP2 enhanced c-Fos expression of POMC neurons; calcium imaging revealed that LEAP2 significantly increases Ca^2+^ concentration in POMC neurons, indicating that LEAP2 can increase the activity of POMC neurons. Future work will focus on the molecular mechanism by which LEAP2 enhances POMC neuronal activity. These data showed that centrally administered LEAP2 reduced food intake and that this correlated with increased POMC neuronal activation in the hypothalamus ARC. In addition, we observed that hM4Di-induced inhibition of POMC neurons blocked the anorexigenic effect of LEAP2. Based on the observation that the hypothalamic POMC expression was stimulated by LEAP2 overexpression in the ARC, we may infer that LEAP2 treatment decreases food intake and body weight by activating POMC neurons and enhancing POMC expression. The currently identified LEAP2 receptor GHSR expresses on both AGRP and POMC neurons, although the expression on POMC is rare. One recent study suggested the suppression of food intake by LEAP2 highly relies on normal GHSR function (54); however, another study stated that LEAP2 functions independently of GHSR (14). Additionally, Chen et al. demonstrated that ghrelin stimulates POMC neurons through an unidentified mechanism that is distinct from conventional GHSR (55). Given the complexity of the biology of GHSR, whether LEAP2 activates POMC neurons through GHSR is uncertain. One report showed that motile sperm domain-containing protein 2 (MOSPD2) is a receptor mediating the LEAP2 effect on monocytes/macrophages in fish (56). Another report described MOSPD2 as a potential therapeutic target for the central nervous system (CNS) inflammation (57). We also agree with the literature, which suggests that future studies are required to investigate the possible interaction between LEAP2 and MOSPD2 (14).

A previous study reported that LEAP2 deletion raised body weight, food intake, lean mass, hepatic fat, and body length in females on long-term HFD compared to WT littermates, which were not observed in HFD-exposed males (12). This discrepancy might be due to the higher level of plasma acyl-ghrelin in HFD-fed female LEAP2-KO mice (12). One study found ghrelin’s effect on feeding is more potent in females (58), while a higher sensitivity to ghrelin’s orexigenic effects was reported in intact males and ovariectomized rats compared to intact females in another study (59). Gender differences exist, and these differences may be associated with the effects of gonadal steroids. Importantly, sex-dependent differences in the regulation of feeding behavior and weight gain have been well described in both males and females in different species (60, 61). In obese and nonobese children, serum LEAP2 levels were higher in girls than in boys (62). Another study on 3~12-year-old children reported no gender differences were found in plasma LEAP2 levels (63). Similarly, nonobese male and female subjects showed a similar value of serum LEAP2 (64). The inconsistency suggests the complexity of sexual dimorphism in the regulation of LEAP2 levels. A more recent experiment showed that peripherally continuous LEAP2 administration by mini-pumps reduced body weight and blood glucose in both calorie-restricted C57BL/6J and GHSR-KO mice, and these effects have no gender difference (14). In this study, we investigated LEAP2’s central anorexigenic effect in male individuals only. This is a limitation. Factors such as sex, age, and metabolic status will have to be considered in future studies.

In summary, this study shows for the first time that long-term overexpression of LEAP2 in the ARC leads to decreases in food intake and body weight under chow and obesogenic conditions. Appetite suppression and lower body weight in LEAP2-overexpressing mice are partially ascribed to the increased expression of POMC. We further demonstrate that LEAP2 enhances POMC neuronal activity and that these neurons mediate the anorexigenic effect of LEAP2. Although the detailed mechanism awaits further elucidation, developing novel strategies that target the upregulation of central LEAP2 expression might provide a promising approach to combat obesity and its associated diseases.

## Data availability statement

The original contributions presented in the study are included in the article/**Supplementary Material**. Further inquiries can be directed to the corresponding author.

## Ethics statement

The animal study was reviewed and approved by the Animal Care and Use Committee of Huazhong University of Science and Technology.

## Author contributions

YL and GC conceived the project and designed the study. GC, HP, NY, YZ, and XL performed the experiments. GC, NY, and YZ analyzed the data. GC wrote the first draft of the manuscript. YL edited and reviewed the manuscript. All authors contributed to manuscript revision and read and approved the submitted version.

## Funding

This work was supported by the National Natural Science Foundation of China (82071508 and 81671331 to YL).

## Acknowledgments

We thank Professor Guo Zhang (Huazhong University of Science and Technology) for providing the POMC-Cre mice and experimental assistance.

## Conflict of interest

The authors declare that the research was conducted in the absence of any commercial or financial relationships that could be construed as a potential conflict of interest.

## Publisher’s note

All claims expressed in this article are solely those of the authors and do not necessarily represent those of their affiliated organizations, or those of the publisher, the editors and the reviewers. Any product that may be evaluated in this article, or claim that may be made by its manufacturer, is not guaranteed or endorsed by the publisher.
