# Supplementary material for: Involvement of POMC neurons in LEAP2 regulation of food intake and body weight
Source: Front Endocrinol (Lausanne). 2022 Oct 28;13:932761. doi: 10.3389/fendo.2022.932761 (PMC9650057; doi:10.3389/fendo.2022.932761)
Supplement: Supplementary file 1 [file DataSheet_1.docx]

Supplementary Material

**
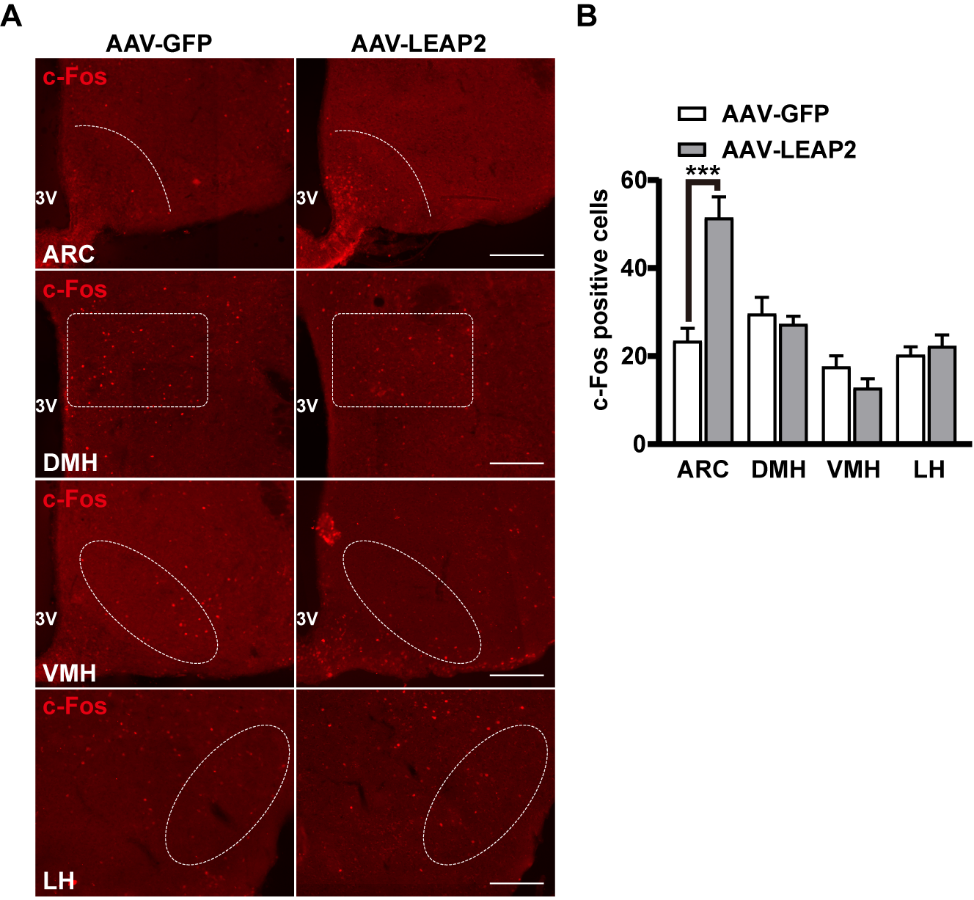
Supplementary Figure 1.** Effects of LEAP2 overexpression in the ARC on neuronal activity in the hypothalamus. **(A)**  Representative images showing the expression of c-Fos in the arcuate nucleus (ARC), dorsomedial nucleus (DMH), ventromedial nucleus (VMH), and lateral hypothalamus (LH) of mice injected with AAV-GFP or AAV-LEAP2 at 4 weeks after virus injection on chow diet. 3V, third ventricle. Scale bars, 200 μm. **(B)** The numbers of c-Fos-positive cells in the ARC, DMH, VMH, and LH. n = 6/group, two-tailed Student’s t-test, *t* = 5.104, *** *p* = 0.0005 < 0.001 for ARC; *p* > 0.05 for DMH, VMH, and LH. Data are presented as mean ± SEM. *** *p* < 0.001.


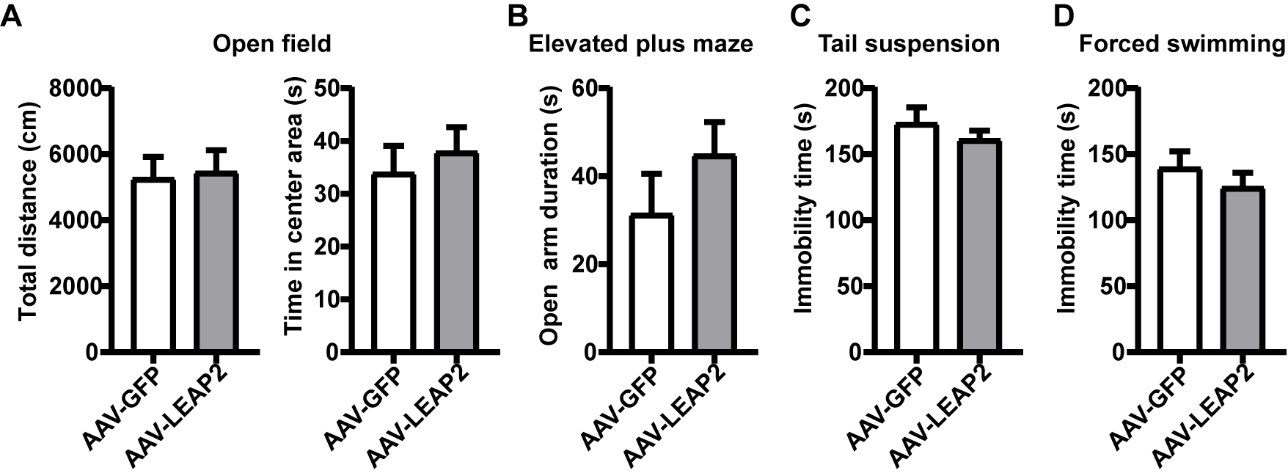


**Supplementary Figure 2.** LEAP2 overexpression in the ARC of mice does not affect animal behaviors. **(A–D)** Open field test (A), elevated plus maze test (B), tail suspension test (C), and forced swimming test (D) after virus injection for 4 weeks in mice fed a chow diet. n = 9–10/group, two-tailed Student’s t-test, *p* > 0.05. Data are presented as mean ± SEM.

**
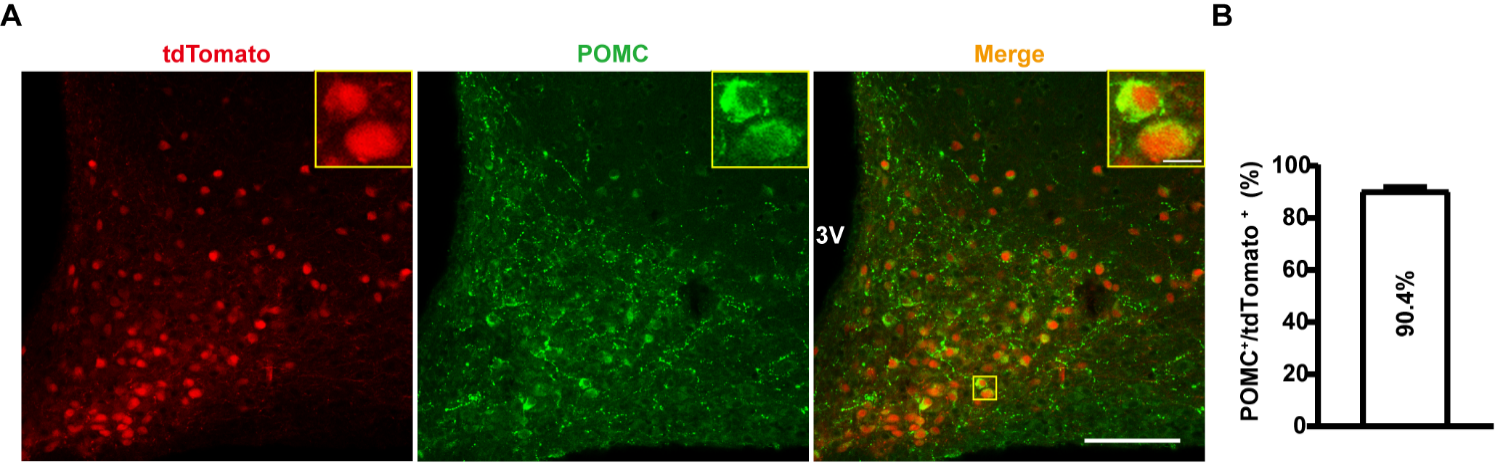
Supplementary Figure 3.** Specificity of the POMC-Cre; Rosa-tdTomato mice. **(A)** Representative hypothalamic section from POMC-Cre; Rosa-tdTomato mice expressing tdTomato and immunostained for POMC. 3V, third ventricle. 100 and 10 μm for the low and high-magnification images, respectively. **(B)** The percentage of tdTomato expressing neurons immunopositive for POMC, n = 5 mice.

**
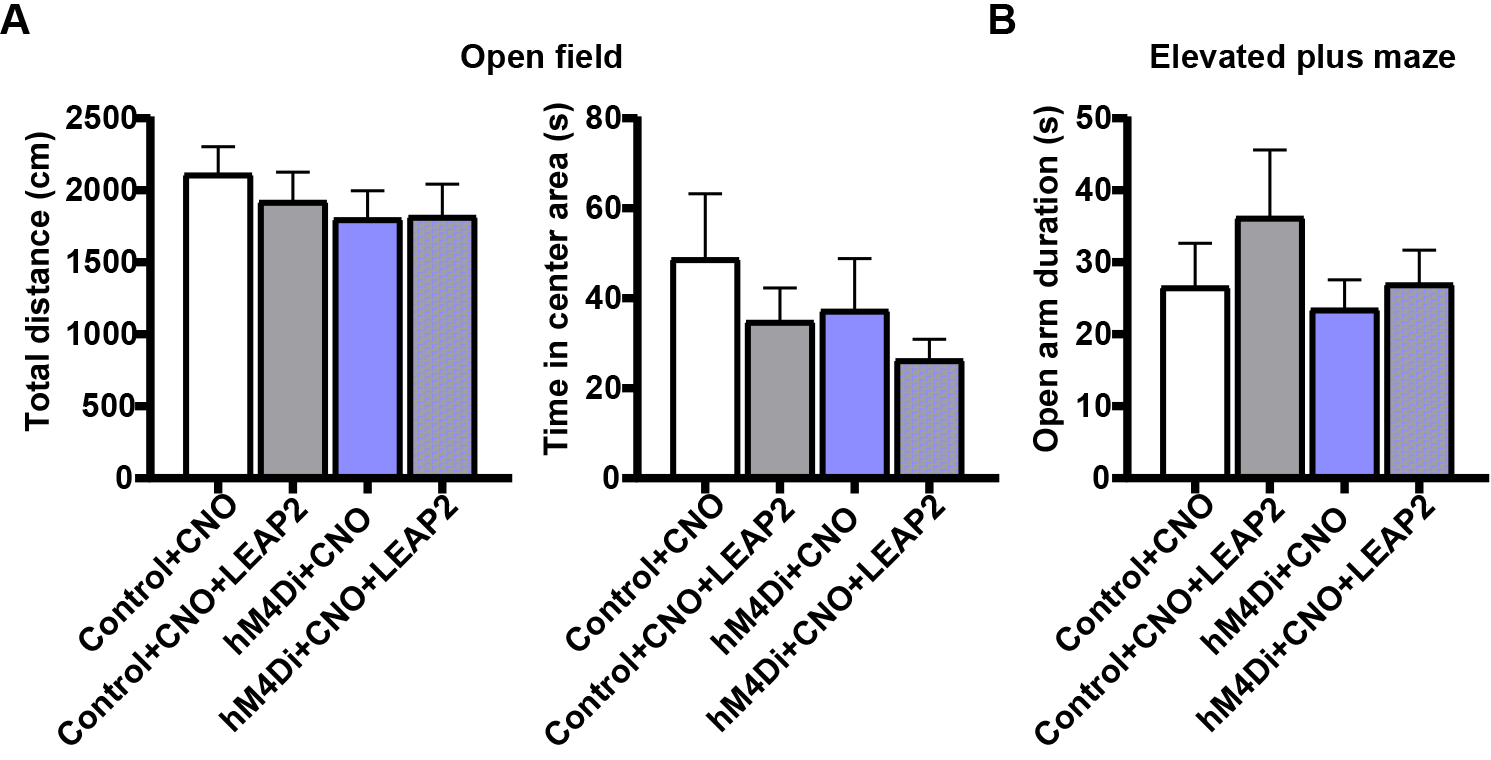
**

**Supplementary Figure 4.** Effects of CNO co-injected with aCSF or LEAP2 on anxiety-like behaviors. **(A, B)** WT or POMC-Cre mice infected with AAV-hM4Di-EGFP in the ARC were injected with CNO (3 mg/kg, i.p.) and received aCSF or LEAP2 (10 nM, i.c.v.), then behavioral tests were conducted 30 min later for open field test (**A**) and elevated plus maze test (B). n = 8/group, one-way ANOVA, *p* > 0.05. Data are presented as mean ± SEM.

**Supplementary Methods**

**Behavioral Tests**

The investigators conducted the behavioral tests unaware of the experimental design during the light period. After a minimum 4-week post-surgery recovery, mice were handled for 5 min twice a day for 3 days and acclimated to the behavior room for 1 h on the day of the experiment. Animal behaviors were video-tracked and analyzed with SuperMaze software (Xinruan Information Technology Co. Ltd., Shanghai, China).

The open-field test was conducted to assess locomotor activity and anxiety-like behavior (1). A 45 × 45 × 45 cm opaque polypropylene box was used as the arena. Every mouse released from the center was allowed to freely explore the arena for 10 min. After each trial, the apparatus was cleaned with 75% alcohol to eliminate the olfactory cues. Total distance traveled and time spent in the center area were analyzed.

The elevated plus-maze test was conducted to evaluate anxiety-like behavior (2). The maze has two open arms and two closed arms (30 cm long, 5 cm wide, and 50 cm high from the floor) extended from a central platform. Each mouse released from the center was allowed to explore the maze for 5 min. Time spent in open and closed arms in the maze was analyzed.

The tail suspension and forced swimming tests were used to evaluate despair-like behavior (3). For the tail suspension test, mice were gently suspended approximately 50 cm above the table by the tail with adhesive tape to a hook for 6 min. For the forced swimming test, mice were gently released into a transparent and vertical Plexiglas cylinder (30 cm height × 10 cm diameter) filled with 23–25°C water up to a depth of 20 cm for 6 min. Total immobility time of the last 5 min in the two tests was analyzed.

**Immunofluorescence staining**

c-Fos expression in the hypothalamus nuclei was assessed 4 weeks after the last injection of AAV-GFP or AAV-LEAP2. The primary antibody and the second antibody were rabbit anti-c-Fos (1:500, ab214672, Abcam) and Alexa Fluor 594–conjugated goat anti-rabbit IgG (1:250, SA00006-4, Proteintech), respectively.

To examine the identity of the labeled tdTomato^+^ cells in the ARC, POMC-Cre; Rosa-tdTomato mice were perfused and brain sections were prepared. POMC immunostaining was performed in the sections containing ARC. The primary and secondary antibody were rabbit anti-POMC (1:300, BM5411, BOSTER) and Alexa Fluor 488–conjugated goat anti-rabbit IgG (1:250, AS053, ABclonal), respectively. 20 × confocal images were taken and the number of neurons expressing tdTomato or POMC was counted.

**References**

1. Ogrodnik M, Zhu Y, Langhi LGP, Tchkonia T, Krüger P, Fielder E, et al. Obesity-Induced Cellular Senescence Drives Anxiety and Impairs Neurogenesis. *Cell Metabolism* (2019) 29(5). doi: 10.1016/j.cmet.2018.12.008.

2. Walf AA, Frye CA. The Use of the Elevated Plus Maze as an Assay of Anxiety-Related Behavior in Rodents. *Nat Protoc* (2007) 2(2):322-8.

3. Palucha-Poniewiera A, Podkowa K, Rafalo-Ulinska A, Branski P, Burnat G. The Influence of the Duration of Chronic Unpredictable Mild Stress on the Behavioural Responses of C57bl/6j Mice. *Behav Pharmacol* (2020) 31(6):574-82. Epub 2020/05/21. doi: 10.1097/FBP.0000000000000564.
